# Outlines of Practical Physiological Chemistry

**Published:** 1892-06

**Authors:** 


*Outlines of Practical Physiological Chemistry.* By F. CHARLES LARKIN, F.R.C.S., and RANDLE LEIGH, M.B., B.Sc. (Lond.). Second Edition. Pp. 86. London: H. K. LEWIS. 1891.

This little book is well arranged, and bears internal evidence that it has been compiled by men who are accustomed to teach the subject, and have a thorough acquaintance with the needs of medical students. We think that the special aim of the book has been attained; namely, "to bring into prominence those portions of the subject which actual daily experience of medical practice has taught us will be most useful to the student in the practice of his profession in after life." As it also satisfactorily deals with the chief pathological constituents of urine, and the examination of the fluids of the body, we are sure that the authors are justified in the opinion they further express, that the "work will be of service to medical practitioners as a handy guide to the examination of various physiological and pathological substances."

